# A Review of Exercise as Medicine in Cardiovascular Disease: Pathology and Mechanism

**DOI:** 10.14336/AD.2019.0516

**Published:** 2020-03-09

**Authors:** Piotr Gronek, Dariusz Wielinski, Piotr Cyganski, Andrzej Rynkiewicz, Adam Zając, Adam Maszczyk, Joanna Gronek, Robert Podstawski, Wojciech Czarny, Stefan Balko, Cain CT. Clark, Roman Celka

**Affiliations:** ^1^Laboratory of Genetics, Department of Dance and Gymnastics, Poznań University of Physical Education, Poznań, Poland.; ^2^ Department of Anthropology and Biometry, Poznań University of Physical Education, Poznań, Poland.; ^3^Department of Cardiology and Cardiosurgery, I^st^ Cardiology Clinic, City Hospital in Olsztyn, University of Warmia and Mazury in Olsztyn, Poland.; ^4^Department of Sports Training, The Jerzy Kukuczka Academy of Physical Education in Katowice, Katowice, Poland.; ^5^Department of Methodology and Statistics, The Jerzy Kukuczka Academy of Physical Education in Katowice, Katowice, Poland.; ^6^Department of Physical Education and Sport, University of Warmia and Mazury in Olsztyn, Olsztyn, Poland.; ^7^Department of Human Sciences, University of Rzeszow, Rzeszów, Poland.; ^8^Department of Physical Education and Sport, Faculty of Education, Jan Evangelista Purkyne University in Usti nad Labem, Czech Republic.; ^9^School of Life Sciences, Coventry University, Coventry, CV1 5FB, United Kingdom.

**Keywords:** physical activity, longevity, cardiovascular disease, public health problem, aging

## Abstract

**Background:**

Physical inactivity and resultant lower energy expenditure contribute unequivocally to cardiovascular diseases, such as coronary artery disease and stroke, which are considered major causes of disability and mortality worldwide.

**Aim:**

The aim of the study was to investigate the influence of physical activity (PA) and exercise on different aspects of health - genetics, endothelium function, blood pressure, lipid concentrations, glucose intolerance, thrombosis, and self - satisfaction. Materials and

**Methods:**

In this article, we conducted a narrative review of the influence PA and exercise have on the cardiovascular system, risk factors of cardiovascular diseases, searching the online databases; Web of Science, PubMed and Google Scholar, and, subsequently, discuss possible mechanisms of this action.

**Results and Discussion:**

Based on our narrative review of literature, discussed the effects of PA on telomere length, nitric oxide synthesis, thrombosis risk, blood pressure, serum glucose, cholesterol and triglycerides levels, and indicated possible mechanisms by which physical training may lead to improvement in chronic cardiovascular diseases.

**Conclusion:**

PA is effective for the improvement of exercise tolerance, lipid concentrations, blood pressure, it may also reduce the serum glucose level and risk of thrombosis, thus should be advocated concomitant to, or in some cases instead of, traditional drug-therapy.

Daily physical activity (PA) patterns were established not in an exercise laboratory, but by the natural need of humans to move and stimulate the cardiovascular system. Our genetically determined biology evolved during the hunter - gatherer period when adapting to environmental stressors, and thus helped define the human genome [1]. However, analysis of mitochondrial DNA indicates that the genetic constitution of contemporary humans has remained relatively constant for over 50 millennia, and the portion of the human genome that determines our basic anatomy and physiology has remained almost unchanged over the past 40,000 years [2]. It has been estimated that, historically, seeking food and water resulted in an energy expenditure of approximately 1,000 to 1,500 kcal/day [3]. Individuals that developed adaptive mechanisms for an increased energy expenditure had increased chances of survival. These adaptive mechanisms were based mostly on the optimization of intracellular metabolism, favourable cooperation of multiple organs, improvement of contractile function, power of locomotion and life-sustaining processes [4]. Contemporary knowledge dictates that daily PA substantially alters the expression of a substantial proportion of genes that comprise the human genome [5,6]. Since from an evolutionary genetic perspective, metabolism of contemporary living people relies on the mechanisms which have not changed for years, it may be better that current PA levels should, to some extent, reflect those of our hunter - gatherer ancestors.

Exercise-induced modifications of gene expression result in rapid but transient improvement, not only including cardiovascular parameters [e.g., blood pressure, lipid metabolism, resting heart rate (HR), thrombosis events, glucose intolerance, autonomic balance], but also on musculoskeletal, pulmonary; sleep quality; mood; immunity and consequently general fitness [7]. Furthermore, basic compound structures of chromosomes (telomeres) may also be influenced by exercise. It is known that telomere length shortens with age, and such progressive shortening of telomeres may result in to senescence, apoptosis, or oncogenic transformation of somatic cells, influencing the health and longevity of an individual [8]. In a previous review, Shammas [9] noted that shorter telomeres have been significantly associated with increased incidence of diseases and mortality. Shammas further asserted that the rate of telomere shortening could be either increased or decreased by specific lifestyle factors, particularly in relation to achieving adequate levels of PA, offering the potential to reduce the rate of telomere shortening or at least prevent excessive telomere attrition, leading to delayed onset of age-associated diseases and increased lifespan [9].

With regards to the expenditure of energy, today’s western lifestyle results in only 38% of the energy expenditure relative to body mass compared to our ancestors’ average accrual [7]; whilst even greater discrepancy exists for total energy expenditure (TEE). To shed practical light on these values, the average person would need to expend and extra 72kj of energy per kilogram of body mass per day (~17 kcal/kg/d) to achieve the TEE of hunter-gatherers: the equivalent of a 19 km (12 mile) walk every day for a 70 kg man.

Based on the significance of PA in health and well-being and the need to understand the potential association between exercising and other intrinsic factors, the purpose of this study was to conduct a narrative review to investigate the influence of PA and exercise on cardiovascular system, risk factors of cardiovascular diseases and to discuss possible mechanisms of this actions.

## MATERIALS AND METHODS

The content of this review article is based on a narrative literature review conducted using online databases; Web of Science, PubMed and Google Scholar in the period from database inception to March 2019. In our literature review, we focused especially on the effects of PA and exercise on cardiovascular disease, including genetic factors, mechanism of action and intrinsic factors. Search keywords included: “health” OR “physical activity” OR “exercise” OR “lifestyle factors” OR “longevity” OR “diet” OR “body weight” AND “telomere length” OR “blood pressure” OR “heart rate” OR “cardiovascular disease”. All key search terms were combined, using Boolean logic, such that one term broadly relating to health, one term related to physical activity/exercise and one term related to mechanism, genetics or prevention, was searched, and subsequently, narratively reviewed.

Briefly, a narrative review endeaours to summarize different primary studies from which conclusions may be drawn into an integrated interpretation [10, 11],. Results are of a qualitative, as opposed to a quantitative nature and accepted to facilitate extended understanding within a field [12]. For this review, we adopted the methodological rhetoric of Greenhalgh et al [13] to narrative reviews. Greenhalgh et al [13] assert that when trying to make sense of a complex topic, it is imperative to review literature from multiple sources and from diverse disciplines. The standard approach to critiquing a body of knowledge is to view the work across four broad perspectives: conceptual (what counts as a legitimate problem within this field); theoretical (how the things studied relate to one another in the world); methodological (how the problem is investigated); and instrumental (the tools and techniques used to understand the concept(s) more clearly in the real world). The argument Greenhalgh et al [13] propose around narrative review is that, when confronted with multiple perspectives, the narrative is created through the process of having to traverse conceptual, theoretical, methodological, and instrumental boundaries to create coherence and a more integrated understanding of the topic under scrutiny. The theoretical question facing our team centred around what influence PA may have on cardiovascular disease, with our narrative focussed on, and segmented into, the following sections; telomeres, vascular endothelium, thrombosis, blood pressure, glucose intolerance, blood lipids, and physical activity and its limitations.

## RESULTS AND DISCUSSION

### Telomeres

Telomeres, the specific DNA-protein structures found at both ends of each chromosome, protect genome from nucleolytic degradation, unnecessary recombination, repair, and inter-chromosomal fusion. Telomeres therefore play a vital role in preserving the information in our genome and are linked to human longevity [14]. With increasing age, and successive cell division, telomeres undergo incomplete replication leading to gradual shortening [15], as a consequence of its attrition, increases the risk of cancer and cell aging simultaneously emerge [16].

As the telomere length and its shortening over time vary among individuals, it is believed to be stable from childhood but may begin to decline in older adulthood [17]. In humans, average telomere length diminishes from 11 kilobases after birth [18] to less than 4 kilobases at an older age [19], however to a greater extent in men than women [20]. Njajou et al [21] observed that leukocyte telomere length is positively associated with the number of years of healthy living, which may indicate that leukocyte telomere length is a biomarker for healthy aging. Thus, a shorter telomere is suggested to be older than longer ones [22].

It is empirically and consistently demonstrated that physical conditioning is associated with healthy aging while lowering the risks of a number of chronic diseases [23]. However, the relationship between telomere length and the level of physical training (intensity and duration/volume measured by kcal burned to exercises per week - kcal/wk) remains somewhat equivocal.

Hassett et al [24] support a link between premature cellular aging and chronic pain as the leukocyte telomere length (TL) is a measure of cellular aging. Investigation of relationship between chronic pain and telomere length on a group of 66 women with fibromyalgia and 22 female healthy controls showed that higher levels of pain within fibromyalgia were significantly associated with shorter telomere length (p = 0.039) [24]. When pain and depression were combined, patients categorized as high-pain/high-depression had an age-adjusted telomere length 265 base pairs shorter than those with low - pain/low - depression (p = 0.043 [24]. The authors [24] reported a correlation between telomere length and pain threshold/sensitivity and grey matter volume, such that patients with shorter telomeres were more sensitive to evoked pain and had less grey matter in brain regions associated with pain processing [24]. Comparable results were reported by Sibille et al [25] when assessing the relationship of chronic pain and perceived stress with cellular aging on a group of 36 subjects between the ages of 47 and 75y. In this study, 18 individuals presented with current knee pain, experienced chronic pain, and were confirmed to have radiographic knee osteoarthritic changes without rheumatoid arthritis, heart disease or uncontrolled medical conditions including high blood pressure, diabetes, and gout.

Ludlow et al indicated that moderate PA levels may provide a protective effect on peripheral blood mononuclear cells telomere length compared with both low and high exercise energy expenditure levels [26]. Studies examining the relationship between physical exercise and telomere length in skeletal muscle cells and leukocytes are summarized in Table 1.

A conclusion may be drawn that the consequence of higher physical training levels, whether aerobic or resistance is longer leukocyte or skeletal muscle telomere length in comparison to a sedentary lifestyle. What level of workload and what duration/volume per week is positive and what is negative to telomere length? The answer may be found in results of Ludlow et al. [26], where moderate levels of physical effort [991-2340 kcal/wk: 2nd and 3rd quartile] proved to be more beneficial in protecting telomere shortening of exercise energy expenditure than 1st quartile of exercise energy expenditure (0 - 990 kcal/wk) and 4th quartile (>3540 kcal/wk); where shorter peripheral blood mononuclear cell (PBMC) telomere length was observed.

However, within the literature exists examples of over-interpretation, and as such, should be carefully interpreted by readers. Arsenis et al [39] asserted Ludlow et al. [26] and Werner et al. [40] have found positive correlations between telomere length and aerobic fitness (VO2 max) [39].

Whilst some inappropriate reporting may be evident, this should not abate the crucial relationship of physical activity/inactivity for telomere length and as the consequence human longevity. However, attention must be given to the large range of different exercise protocols (e.g., volume and intensity (workload) of physical exercise), measured cell types, self - reported activities and exercise time duration. Although it may look that interventional studies do not seem to support thesis about direct PA impact on telomere length, it is apparent that changes in telomere length, even after few months of exercise training, may still not be evident [39, 41]. Accordingly, it is advisable that measures of leukocyte telomeres should not be obtained immediately following vigorous activity, as after a single maximal exercise session there may be and overlap of cell recruitment with a greater history of replication [42].

**Table 1:** Effect of exercise on telomere length.

Participants	Type of Exercise	Influence on Telomere Length	Ref
123 males (56 healthy non - maraton runners, 67 ultra - maraton runners)	Ultra - maraton running, exercise 40 - 100 km/week, ?2 years	Longer leukocyte telomere length in runners *vs.* non - runners	[27]
62 adults (20 athletes, 42 sedentary controls)	Endurance exercise	Longer salivary telomere length in endurance athletes *vs*. sedentary control	[28]
2401 white twin adults	Self - reported physical activity (4 groups based on physical activity levels)	Longer leukocyte telomere length with increasing exercise level	[29]
69 healthy adults	Various aerobic exercise (divided into quartiles based on exercise energy Expenditure: 0-990, 991-2340, 2341-3540, and 93541 kcal/wk)	Longer leukocyte telomere length in 2nd quartile *vs.* 1st and 4th quartile. Same telomere length in 2nd quartile *vs*. 3rd quartile.	[30]
44 healthy postmenopausal women (21 sedentary subjects, 23 habitual exercise participants)	Aerobic and resistance exercise for 60± minutes, >3 times per week, for > 12 months	Longer leukocyte telomere length in aerobic and resistance exercise participants vs sedentary subjects.	[31]
14 healthy adults (7 non - lifters, 7 power lifters)	Power lifting; 8±3 years	Longer skeletal muscle telomere length in power lifters vs non - lifters.	[32]
20 young and older men (10 medium activity level, 10 endurance athletes).	Endurance exercise (long distance skiing & track running competitions); Medium activity (moderately physically active)	Longer skeletal muscle telomere length in older athletes vs older medium - activity individuals. Same telomere length in young athletes vs young medium - activity individuals.	[33]
7,813 adult women	Eight possible physical activities	Increase in leukocyte telomere length (0.10-SD) was observed when comparing the most to the least active women.	[34]
944 adults with stable coronary heart disease	Self - reported physical activity	Shorter telomere length associated with physical activity, but not after multivariate adjustment.	[35]
32 adults (15 sedentary healthy subjects, 17 marathon runners)	Marathon running, 32±9 miles/week, 14±11 years	Same leukocyte telomere length in runners *vs*. sedentary subjects	[36]
37 adults (19 sedentary subjects, 18 endurance runners)	Endurance running: 40 km/week, ?7 years	Same (skeletal muscle) telomere length in runners *vs.* sedentary individuals. Shorter telomere length in subjects with longer exercise history *vs*. shorter training history. Shorter telomere length in subjects with greater volume of training hours *vs.* lower volume of training hours.	[37]
25 healthy young and 32 older adults	Vigorous aerobic exercise ?5 days/week, >45 min/Day, ?5 years	Same leukocyte telomere length in older athletes vs. older sedentary subjects.	[38]

There are several potential mechanisms explaining how exercises may affect telomere length. Training leads to up-regulation of telometric repeat-binding factor 2 (TRF2), that plays a role in protecting telomeres from shortening [43]. In the middle-aged individuals it was also found, that in response to exercise, there is up-regulation of Ku proteins (which constitute the DNA repair pathway). Exercise training contributes to reduced levels of inflammatory markers in individuals with enhanced chronic inflammation [44, 45], and it has been shown that inflammation related proteins like tumor necrosis factor alpha (TNF-alpha), interleukin 6 (IL-6), nuclear factor kappa B, poly (ADP-ribose) polymerase 1, repressor-activator protein 1 and telomerase reverse transcriptase may be involved in telomere shortening process [46, 47]. Chronic exercise may lower oxidative stress and therefore protect telomeres from shortening inflicted by excessive reactive oxygen species (ROS) [48]. Moreover, physical training stimulates the satellite cells (skeletal muscle cell precursors), which counteracts the decline of satellite cells that occurs with aging. The quantity of satellite cells in turn is positively correlated with the skeletal muscle telomere length [49].

Summarizing, low PA may be related the shortening of telomere size and, consequently, reducing longevity. Therefore, attaining global PA is advisable.

### Vascular endothelium

Vascular endothelium is an active organ that is indispensable for the maintenance of vascular homeostasis, regulation of vascular tone, acting paracrine, endocrine, and autocrine, active transport of substrates, what makes it crucial for normal and regular cardiorespiratory system function. However, since the normal endothelial functioning is necessary for health, development of vascular endothelial dysfunction accompanying aging is attributed to increased risk of CVD’s occurrences, which, in part, is due to impaired EDD (endothelium - dependent dilation) [49, 50, 51].

Appropriate vasodilation of blood vessels is, among other factors, due to secretion of nitric oxide (NO) endothelium - derived relaxation factor [52] synthesized from arginine by a family of three distinct isoforms of nitric oxide synthase (NOS) enzymes [53]. The continuous secretion of NO is involved in regulating basal vascular tone, which is a balance between constrictor and dilator influences [54]. NO also plays an important role in the reparation and regeneration of myocardium [55] and in the modulation of oxygen consumption in the myocardium [56]. The endothelial nitric oxide synthase (eNOS or *NOS3*) is encoded by the *NOS3* gene (localization: 7q36) and as the result of extensively screening for polymorphisms, several polymorphic sites have been identified among others: single nucleotide polymorphisms (SNP): -786T/C (rs2070744), G894T (Glu298Asp, rs1799983), as well as the intron 4 variable number tandem repeat (VNTR) [57].

The eNOS gene variants have been extensively investigated to ascertain their relevance/association in increasing susceptibility to cardiovascular disease as well as in the onset of other complex diseases, such as type 2 diabetes, insulin resistance and cancer [58, 59, 60, 61, 62, 63].

Notably, regular endurance exercise is effective for maintaining overall vascular regularity (health) concomitant to aging [64, 65]. Training individuals have been shown to have significantly higher eNOS expression and phospho-eNOS (phosphatidylinositol 3-kinase (PI3K)/Akt-dependent phosphorylated eNOS at Ser1177) levels which was correlated with endothelial function [66]. Extended periods of exertion result in reduced ROS production and contributes to improved bioavailability of NO [67]. Constant exertion has been proved to modulate the expression of miR-221, miR-92a, miR-21 and miR-17 resulting modulation of oxidative and antioxidative enzymes, higher NO production, and better endothelial function [67]. Regular aerobic exercise can prevent the age-associated loss in endothelium-dependent vasodilation and restore levels in previously sedentary middle aged and older healthy men, which represents an important mechanism by which regular aerobic exercise lowers the risk of cardiovascular disease in this population [68].

### Thrombosis

Vascular endothelial cells (ECs) maintain the dynamic balance between immune response functions and anticoagulation [69]. Endothelial dysfunction is associated with thrombosis, and share many common risk factors, such as obesity, diabetes, low PA, smoking, hypertension, and hyperlipidemia [70]. Although head-to-head comparison of exercise and drugs influence on thrombosis is implausible (given cessation of medicine is clinically not recommended), longitudinal studies have shown that low fitness raises thrombosis - related cardiovascular events, e.g., nonfatal myocardial infarctions, strokes, in people with [71] or without a history of CVD [72] and peak exercise oxygen consumption (VO_2_max peak) can be used as accurate predictors of future fatal cardiac events, beyond that predicted by many conventional risk factors [71].

Of particular importance, individuals recovering post coronary stenting are assumed to have an increased risk of stent thrombosis (ST). In a prospective observational study (n=3,672), where patients participated in aerobic exercise training (ET) group (n=1.592) or control group (n=2.080) post successful coronary stenting [73]. The authors [73] observed that although the incidence of ST and major adverse cardiovascular events (MACE), including stroke, myocardial infarction and death, were similar in both groups (1.8% vs. 2.0%, p=0.73, 14.9% vs. 15.0%, p=0.97, respectively); unscheduled hospital visits were significantly lower in the ET group (20.2% vs. 27.2%, p<0.0001). It was asserted that aerobic exercise training (3 times per week or more in sessions of at least 30 min, with exercise intensity that was between “relatively easy and slightly tiring”, and after charge walking) is effective in the prevention of unscheduled hospital visits for worsening angina (HR 0.67, adjusted p<0.0001).

Exercise evokes significant transient responses in both the coagulation and fibrinolytic systems. Directly after exercise an increased levels of platelet factor 4, prothrombin fragment 1 + 2, thrombin-antithrombin complex, tissue plasminogen activator (t-PA), and decreased levels of plasminogen activator inhibitor-1 (PAI-1) activity have been reported. Although the more transient nature of the fibrinolytic response may induce a disturbed balance and contribute to the increased cardiovascular risk (observed shortly after strenuous exercise), habitual PA is associated with enhanced endothelial function (potentially mediated by a platelet-inhibiting effect of NO and prostacyclin) and inversely associated with resting fibrinogen, factor VIII, and von Willebrand factor level, conducing overall a beneficial effect of PA on the risk of VTE incident [74].

In summary, physical conditioning has the potential to positively influence the occurrence, and re-occurrence, of thrombosis-related cardiovascular events, and associated hospital visits.

### Blood pressure

Hypertension (HTN) is a significant risk factor for adverse cardiovascular events including; heart failure, stroke, myocardial infarction, and renal failure. Regular PA and exercise is considered fundamental in the prevention and management of HTN [75, 76]. Every third European is diagnosed with HTN, and it is estimated that in the year 2025, *1 billion and 250 million* people will be diagnosed with hypertension [77]. Direct and indirect costs of HTN amounted to $46.4 billion in 2011 and projections of six - fold increases by 2030 have been proposed; thus, the importance of low - cost non-pharmacological interventions is essential [77].

Exercise training affects blood pressure mostly by the change of vascular function and structure eventually leading to decreased peripheral resistance [78, 79]. Vasodylatation results from improved expression and activation of endothelial NO synthase, superoxide dismutase and improved vascular antioxidant capacity [80, 81]. Decreased vasoconstrictor tone secondary to exercise training mainly occurs through decreased endothelin-1 endogenous bioavailability [82] and also decreased expression of the angiotensin II type 1 receptor (AT1R) [83]. Exercising may influence the diameter of the artery [84], intima media thickness, artery stiffness and compliance [85], and baroreflex sensitivity [86] resulting in BP reduction.

It is currently implausible to directly compare the effects of intense PA or exercise to antihypertensive drugs, since no current studies are available comparing the effects of BP lowering drugs vs. placebo and exercise. Previous meta - analysis [87] (comprising 72 trials, 105 study groups, and 3936 participants) concerning the effects of chronic dynamic aerobic endurance training on BP (resting and ambulatory BP, BP-regulating mechanisms, and concomitant cardiovascular risk factors) highlighted that aerobic endurance training decreases blood pressure (reductions of resting BP (3.0/2.4 mm Hg, p<0.001) and daytime ambulatory BP (3.3/3.5 mm Hg, P<0.01) through a reduction of systemic vascular resistance (decrease by 7.1%, p<0.05). Mechanistically, the sympathetic nervous system (decrease of plasma norepinephrine by 29%, P<0.001), and the renin - angiotensin system (decrease of plasma renin activity by 20%, *P*<0.05), appear to be involved, and favourably affects concomitant cardiovascular risk factors [87]. Data concerning lowering of BP after regular exercise are equivocal, with other studies suggesting even lower values of BP [88, 89].

Additionally, it has been recently established that initiation of hypertension treatment should be based on drug combination [90]. An effective reduction in blood pressure induced by PA may help to avoid effects of polypragmasy e.g. adverse metabolic effects including worse insulin sensitivity and glucose intolerance that contribute to increased risk of new onset diabetes and progression of existing diabetes [91, 92, 93].

In summary, though infeasible to rigorously examine, the effects of physical training or exercise on BP are likely to be comparable to administering common multi-drug combinations, and potentially higher than effect of monotherapy in lowering BP [94].

### Glucose intolerance

The impact of training on glucose metabolism is extensively documented. The main mechanisms include insulin-mediated glucose uptake [95, 96], post-receptor insulin signalling [97], increased transport of glucose to the muscles due to enlarged muscle capillary network and up-regulation of glucose transporter (GLUT4) [98, 99]. The progression of chronic hyperglycemia, a common health aging problem, causes severe metabolic homeostasis alterations, resulting in damage to (non-exhaustively); the retina, peripheral and autonomic nervous systems, cranial, coronary, peripheral vascular trees and kidneys [100]. Regular glycemic control is a standard approach adopted by health care providers, globally, and achieving a hemoglobin A1c (HbA1c) level of less than 7% is a quality measure often used to medical treatment [101], with the aim of lowering HbA_1c_ level to slow the progression of early microvascular disease [102].

It is strongly advocated that only employing medical treatment without accompanying changing lifestyle (diet, PA, exercise, supplements) is not sufficient to abate glucose intolerance. In fact, there is strong evidence that regular exercise, and concurrent utilisation of substrates, is associated with an absolute reduction of 0.67% in glycosylated hemoglobin (HbA1c) levels [95% confidence intervals (CI) 0.49-0.84] [103]. However, upon comparing the efficacy of different training modes, it appears that aerobic exercise is more effective in lowering HbA1c levels (0.73%; 95% CI 0.40-1.06), than resistance (0.57%; 95% CI 0.01-1.14) or combined aerobic and resistance training (0.51%; 95% CI 0.23-0.79).

It is remarkably encouraging that the overall reduction in HbA1c brought about by exercise compares relatively well with drug administration [104, 105].

### Blood lipids

Contemporary challenges for health care systems (HCS) to address is the reduction in coronary atherosclerosis prevalence, total cholesterol (TC), low-density lipoprotein cholesterol (LDL-cholesterol) and triglyceride levels (TG), in addition to other CHD risk factors [106]. Maintenance of low LDL-cholesterol necessitates lower saturated fat and cholesterol intake, where maintenance of a healthy weight, and regular aerobic exercises or combination are front-line interventions for improving lipids and lipoproteins in adults [107, 108]. Although the mechanism of exercise-induced lipid changes is unclear, mechanism may involve increased activity of lipoprotein lipase (LPL) [109], increased expression of ATP-binding cassette transporter A1 (ABCA1) in macrophages [110], increased liver X receptor (LXR) [111] and reduction in plasma level of proproteinase/subtilisin/kexin 9 (PCSK9).

Aerobic exercise interventions statistically significantly change level of TG (-6.0 mg/dl; 95% CI -11.8 to -0.2; -5.7% comparing to baseline value). However, the same effect is not observed in TC (0.9 mg/dl; 95% CI -3.2 to 5.0; +0.4% comparing to baseline value), HDL - cholesterol (1.0 mg/dl; 95% CI -0.2 to 2.1, +2.1% comparing to baseline value), or LDL - cholesterol (2.1 mg/dl; 95% CI -1.5 to 5.7; +1.5% comparing to baseline value) [112]. Recently it appeared that the oxidized LDL concentration in patients with the high training load significantly decreased after 2 years of exercise (41.3 ± 8.6 µmol/L vs 39.4 ± 7.4 µmol/L, respectively) [113]. Nevertheless, comparing exercise vs cholesterol-lowering drugs, particularly the most prevalent statins (simvastatin and atorvastatin), exercise appears far less efficacious [72]. Effects of pharmacotherapy are very evident amounting 29-53% decrease for LDL cholesterol [73, 108, 114, 115].

Concerning exercise as the alternative for drugs application, it should be stated that the current evidence suggests that pharmacological treatment of dyslipidaemia is more efficacious than exercise alone. Notwithstanding, exercise will result in little-to-no side-effects, and therefore warrants consideration.

### Optimal Physical Activity

**Figure 1.**
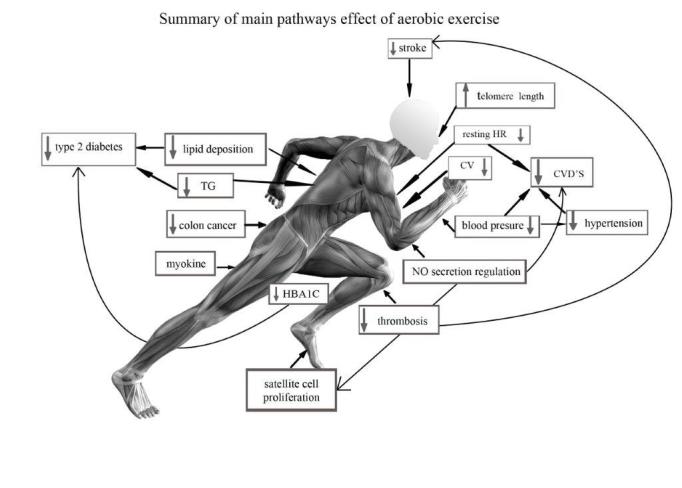
Summary of main pathways effect of aerobic exercise.

In-spite of advances in pharmacology, cardiovascular diseases (CVDs) especially ischaemic heart disease and stroke, are still the main causes of morbidity and mortality, accounting for a combined 15 million deaths globally in 2015 [116, 117]. There is strong epidemiological evidence indicating that the main consequence of regular exercise, in which moderate-to-vigorous intensity is achieved for cardiorespiratory fitness (>8 METs^1^), is the reduction of risks of all-cause mortality [118], CVD, hypertension, stroke, metabolic syndrome, and type 2 diabetes [119] (Figure 1). Individuals who sustain comparably high PA levels tend to live significantly longer than the general population, and have lower mortality rates for both CV disease and cancer [120, 121].

Although, the literature is replete with studies showing that regular PA or systematic exercise confers a favourable protective effect against some chronic disorders and the development or severity [122, 123, 124], the optimal intensity and volume per week, and likewise type of exercise, necessary for maximal protection remains equivocal. This is because each individual is unique in health status (*inter alia* cardiovascular fitness (CRF)), nonetheless in practice it is acceptable to draw some generalization on the basis of specific knowledge especially for healthy, non-smoking persons. The variables that should be taken into consideration are: trainability (the result of CRF level, muscular endurance and strength), risk stratification on the basis of completed medical history and consequently may provide an option of type of exercise and level of intensity that will be optimal for the individual.

Exercise, when planned inadequately to adaptation possibility with too high intensity of chronic aerobic exercise, may lead to a heightened risk of developing atrial fibrillation (AF) [125, 126, 127] or even sudden cardiac death (SCD) in athletes during activities (0.24 episodes per 100,000 athletes - years) [128]. Although it is clear that physical inactivity is a far greater and ubiquitous public health problem than excessive exercise [129]. Vuori et al. [130] even emphasise that exercise is medicine, and therefore it should be seen and dealt with in the same ways as pharmaceuticals and other medical interventions regarding basic and continuing education and training [130].

The current recommendation of PA volume for Americans is at least 150 minutes a week of moderate-intensity, or 75 minutes a week of vigorous-intensity aerobic PA, or an equivalent combination of moderate- and vigorous-intensity aerobic activity [131]. Analysis of multiple studies highlights it may be sensible to limit vigorous exercise to maximum 60 min/d, to facilitate appropriate duration of regeneration and rest [132, 133, 134].

Exercising may effectively increase heart rate variability (HVR) and reduce resting heart rate (RHR) - non - invasive markers of the autonomic nervous system function, ubiquitously used in trials assessing cardiovascular risk [135]. Reduced HRV values (or baroreceptor reflex sensitivity) may be associated with deleterious cardiovascular health and outcomes, whereas elevated RHR is associated with higher mortality [136, 137]. Optimally applied PA as the autonomic nervous system function modulator can contribute to the reduction of cardiovascular risk.

### Self-conscious limitations to PA participation

The current trend in obesity and physical inactivity in Western countries is concerning, whilst nations including Poland, Great Britain, Slovakia, Ireland and Czech Republic have a particularly high prevalence of obesity [138]. Anecdotally, there is a distinct propensity of the general population to prefer medical intervention over physical training and exercise. Clearly, of paramount important task is the changing of long-standing, chronic habitual behaviour.

In examining the difficulties of influencing behavioural characteristics, it has been asserted that human consciousness must be considered [139]. It seemed obvious that level of weekly PA is the result of self - consciousness as well as inactivity (laziness) being opposite to activity, both important personality traits.

It is worth noticing that human technological and social evolution refers between others just to two fundamental personality traits: fear and inactivity laziness, the evolutionary traits, where the need to watch television exactly refers to this phenomenon. In this study the more thematically related seemed inactivity (laziness/idleness) as the dominated and prevalence “first choice” deeply coded algorithm between sit/walk, read/watch, exercise/computer play, keep diet regime/overfeed. Consequences of physical and cognitive laziness constitute significant risk factors for the age-related neurodegenerative diseases [140].

Knowledge that reduction of body mass is the result of higher total energy expenditure (TEE) than daily energy intake seems to be quite common. However, changing self-consciousness of understanding the importance of PA for own life seem much complicated and harder.

Among others, Keefe et al [3] emphasises our attention the instinctive solution to this conundrum is to replicate the PA patterns of our Stone Age hunter-gatherer ancestors [3]. It is as hard as desensitization of human senses accustomed to sodium glutamate and other “supplements” in food. Changing habits is the real-challenge.

### Conclusion

In conclusion, this narrative review highlights that PA and exercise may be effective for the improvement of exercise tolerance, lipid concentrations, blood pressure, glucose intolerance, thrombosis, and self - satisfaction. Exercise is essential for individuals with chronic cardiovascular disease, and should be advocated concomitant to, or in some cases instead of, traditional drug-therapy. However, this should be more intensively researched using randomized controlled trials to confirm the veracity of such holistic assertions.
